# Resurrection of *Hereditas*, a journal with almost 100 years of tradition

**DOI:** 10.1186/s41065-015-0003-8

**Published:** 2015-10-22

**Authors:** Stefan Baumgartner

**Affiliations:** Lund University BMC, D10 22184 Lund, Sweden

For those who followed the fate of *Hereditas* in the last months may have noticed that the journal terminated its activity by the end of 2014. I am extremely happy to announce that *Hereditas* is alive again and as a result of this resurrection, I write this Editorial. I must admit that around 8 months ago, I wrote a similar Editorial for the last issue of *Hereditas* under the wings of the previous publisher, Wiley. At that time, the letter was written in a rather melancholic mood, as the tradition of one of the oldest journals in the world dealing with genetics seemed to come to an end. We should keep in mind that *Hereditas* was founded in 1920 and was thus pretty close to celebrating its 100^th^ anniversary. For this reason, we, the Editors, as well as the owner of *Hereditas*, the Mendelian Society in Lund, are more than delighted to have found BioMed Central as the new partner for continuing our activities and we are looking forward again to serving the scientific community under the new wings of BioMed Central.

## The “old” *Hereditas*

*Hereditas* means almost 100 years of publishing in the field of genetics. During almost one century, *Hereditas* developed its distinct reputation through publishing seminal papers. For example, did you know that the first demonstration of the correct number of human chromosomes (46) was demonstrated through a publication that appeared in *Hereditas* [1]? For a long time, it was thought that the human female chromosome number was 2n = 48 and that of males 2n = 47 [2]. It was Albert Levan and his colleague who in 1956 corrected the number to 46 through his ingenious technique of preparing and staining metaphase chromosomes [1]. The story around the discovery is nicely illuminated by Ulfur Arnason [3]. This 1956 publication had a profound impact on the development of cytogenetics and provided a protocol for preparing chromosomes of such high quality that set the future standard for all cytogeneticists in the world.

## The “new” *Hereditas*

When you compare the “old” Hereditas with the “new” one, then you will notice a number of apparent changes:

- we now offer a wider selection of articles than previously: Research articles (available before), Brief reports (available before), Reviews (available before), Commentaries (new), Letters to the Editor (new), Software (new) and Opinions (new). Hence, authors have a wider selection to choose from, and the likeliness that their manuscript fits into one of these categories is higher. There are also plans to have special issues on topical issues: here, the readership is invited to come up with appropriate suggestions, if they wish.
- *Hereditas* has appointed a second Editor-in-Chief, Professor Yongyong Shi from Shanghai, China, with whom I share the responsibility for the direction and the activities of the Journal. I would like to use the opportunity to warmly welcome Yongyong Shi onboard of *Hereditas*, and I wish him ample satisfaction for this post. Yongyong Shi has vast experience in the genetics of complex traits including mental disorders, tumors or metabolic diseases. Apart from his expertise, he will become a major driving force for our expanding activities in Asia and Australia.
- we now offer a large Editorial Board with eminent scientists (meanwhile more than 40 colleagues).
- the layout of the homepage has been changed: it is now more transparent than previously and all relevant information for authors and readers is readily available therein.
- the homepage is now embedded into the BioMed Central platform.

## Open access: benefits for authors

Publishing with *Hereditas* within BMC also means a plethora of benefits for authors: the most important one to name is “Open access”. Open access means that your article will be available and read by other scientists around the world at no cost and independent of the budget of the librarian. Open access also implies that once your manuscript is accepted, it will be published online immediately. Hence, your data are out, become readily visible and will immediately be listed in PubMed.

Open access also means that you always have online access to your own article, and signifies that authors keep the Copyrights of their work and thus grant anyone the right to reproduce and disseminate their article, provided that it is cited accordingly. Finally, Open access journals have to potential to reach a larger number of readers than subscription-based journals. It is clear that you as the authors will benefit from those facts mentioned above.

## Editorial policy

As mentioned above, *Hereditas* now has an expanded Editorial Board with many excellent scientists at hand. It is our policy that the editorial staff strive for a fast time from submission to final decision on every manuscript submitted to the journal. To this end, we use the well-proven manuscript-submission platform, Editorial Manager. The platform allows authors to upload their manuscript in a intuitive fashion. Later during the reviewing process, the platform allows full transparency for authors to assess the stage of their manuscript. Your manuscript will always be seen by at least 2 reviewers, in some cases we seek the opinion of a third one. We strive for constructive criticism by the reviewers such that authors will receive clear guidance on how to improve the manuscript to ultimately get it accepted. Furthermore, upon receipt of the reviews, authors are given sufficient time to revise their manuscript.

## A wider range of research topics

As far as the research topics are concerned, the “new” *Hereditas* now accepts a wider range of manuscripts than the “old” one. We accept manuscripts across the full scope of genetics and genomics, including those of plants, crops, microorganisms, animals, human diseases and model systems, and, more recently, also manuscripts dealing with novel viral sequences and bioinformatics. It is our goal to provide a wide and interesting selection of articles to readers in future.

It is our intention to celebrate the centenary of *Hereditas*. For this reason, we invite you to publish with *Hereditas*, a journal with almost 100 years of tradition and sharing a distinct reputation.

Stefan Baumgartner, Editor in Chief.
